# Supplementary material for: Acceptability of a COVID-19 Vaccine among the Saudi Population
Source: Vaccines (Basel). 2021 Mar 5;9(3):226. doi: 10.3390/vaccines9030226 (PMC7999879; doi:10.3390/vaccines9030226)
Supplement: Supplementary file 1 [file vaccines-09-00226-s001.pdf]

Acceptability of a COVID-19 Vaccine among the Saudi Population.

Section 1: Sociodemographic characteristics of respondents and general information

---

1.1 ______ **What is your age group?**

______ 18 - 29
______ 30 - 39
______ 40 - 49
______ 50 - 59
______ ≥ 60

1.2 ______ **What is your gender?**

______ Male
______ Female

1.3 ______ **What is your marital status?**

______ Single
______ Married
______ Widowed
______ Divorced

1.4 ______ **What is the highest level of education you have completed?**

______ Primary school
______ Intermediate school
______ High school
______ Diploma before university
______ Bachelor's degree
______ Diploma post-university (postgraduate diploma)
______ Postgraduate degree

1.5 ______ **Please indicate the region you currently reside in:**

______ Riyadh Region
______ Makkah Region
______ Madinah Region
______ Qassim Region
______ Eastern Region
______ Aseer Region
______ Tabuk Region
______ Hail Region
______ Northern Borders Region
______ Jazan Region
______ Najran Region
______ Al Baha Region
______ Al Jowf Region

1.6 ______ **What is your current occupation?**

______ Government employee
______ Non-government employee
______ Self-employed
______ Student

______
______ Retired
______ Unemployed

1.7 ______ **What category best fits your overall monthly income?**
______ < SR 3000
______ SR 3000 to less than 5000
______ SR 5000 to less than 7000
______ SR 7000 to less than 10,000
______ SR 10,000 to less than 15,000
______ SR 15,000 to less than 20,000
______ SR 20,000 to less than 30,000
______ ≥ SR 30,000

Section 2: Health status, vaccination history, and perceived risk of COVID-19

2.1 ______ **Do you suffer from any chronic diseases?**
______ Yes Please specify ……………………………………………….
______ No

2.2 ______ **Have you been vaccinated for the seasonal flu in the past?**
______ Yes
______ No

2.3 ______ **Have you ever refused a vaccine recommended by a physician because of doubts about it?**
______ Yes
______ No

2.4 ______ **To what extent do you think coronavirus poses a risk to people in Saudi Arabia?**
______ No risk at all
______ Minor risk
______ Moderate risk
______ Significant risk
______ Major risk

2.5 ______ **Have you been (or currently) infected with COVID-19?**
______ Yes *If "yes", please go to Q 2.7*
______ No

2.6 ______ **How concerned are you with being infected with COVID-19?**
______ Very high
______ High
______ Fair
______ Low
______ Very low

2.7 ______ **Have any of your family members been (or currently) infected with COVID-19?**
______ Yes
______ No

Section 3: Acceptability of a COVID-19 vaccine

3.1 Scientists around the world are currently working on a vaccine that could prevent people from getting infected with COVID-19. It is hoped that the vaccine will become available in a few months. In the case that a COVID-19 vaccine becomes available in the next few months, with an effective rate between 90-94.5%. Would you be willing to get the COVID-19 vaccine if it was provided free by the government?

______ Yes

______ No

3.2 Do you support that the COVID-19 vaccine should be compulsory for all citizens and residents in Saudi Arabia?

______ Yes

______ No

Thank you for your time in participating in this study
